# Communic Acids: Occurrence, Properties and Use as Chirons for the Synthesis of Bioactive Compounds

**DOI:** 10.3390/molecules17021448

**Published:** 2012-02-06

**Authors:** Alejandro F. Barrero, M. Mar Herrador, Pilar Arteaga, Jesús F. Arteaga, Alejandro F. Arteaga

**Affiliations:** 1 Departamento de Química Orgánica, Facultad de Ciencias, Instituto de Biotecnología, Universidad de Granada, Campus de Fuente Nueva, s/n. 18071 Granada, Spain; Email: arteagaburon_pilar@hotmail.com; 2 Departamento de Ingeniería Química, Química Fisíca y Químíca Orgánica, Facultad de Ciencias Experimentales, Universidad de Huelva, Campus el Carmen, s/n, 21071, Huelva, Spain; Email: jesus.fernandez@diq.uhu.es; 3 Departamento de Ingeniería Química, Facultad de Ciencias, Universidad de Granada, Campus de Fuente Nueva, s/n. 18071 Granada, Spain; Email: jandro@ugr.es

**Keywords:** communic acids, labdanes, bioactivity, synthesis, chirons

## Abstract

Communic acids are diterpenes with labdane skeletons found in many plant species, mainly conifers, predominating in the genus *Juniperus* (fam. *Cupresaceae*). In this review we briefly describe their distribution and different biological activities (anti- bacterial, antitumoral, hypolipidemic, relaxing smooth muscle, *etc.*). This paper also includes a detailed explanation of their use as chiral building blocks for the synthesis of bioactive natural products. Among other uses, communic acids have proven useful as chirons for the synthesis of quassinoids (formal), abietane antioxidants, ambrox and other perfume fixatives, podolactone herbicides, *etc.*, featuring shorter and more efficient processes.

## 1. Introduction

**Figure 1:**
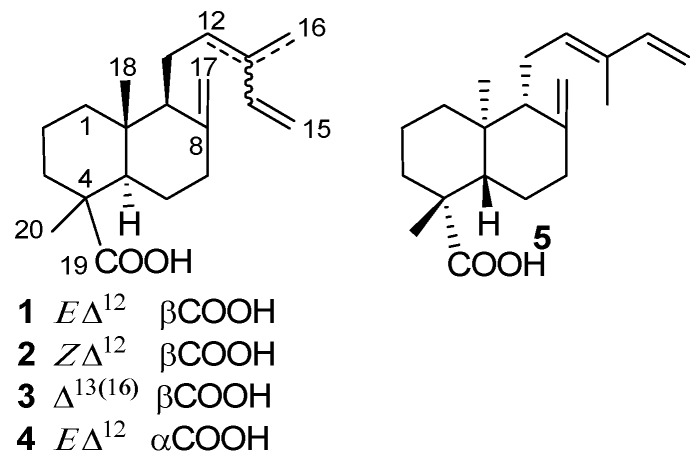
Structure of the communic acids.

Communic acids are a group of diterpenic natural products [1,2,3,4] with a labdane skeleton containing three double bonds and a carboxyl group at position 19 (Figure 1). Five communic acids have been described to date that differ in the location of the double bonds and the orientation of the carboxyl group: *trans*-communic acid (**1**) with the double bonds located in positions 8(17), 12 and 14, with Δ^12^ double bond *E* stereochemistry, and axial carboxyl group orientation, *cis*-communic acid (**2**) the *Z* isomer of the former, *mirceo*communic acid (**3**), also named *iso*communic acid, regioisomer of the former, where the Δ^12^ double bond moves to Δ^13(16)^, 4-*epi*-*trans*-communic acid (**4**), a C4 epimer of **1** and *ent*-*trans*-communic acid (**5**) is the (−) enantiomer of **1**. Of these, the most abundant in Nature is **1**.

## 2. Sources

Communic acids are widely distributed in *Cupresaceae* species, especially in the genus *Juniperus*. Although there are species that contain several of them, the most common case is the presence of only one. A tertiary mixture of **1**–**3** is found in *Juniperus nana* Willd. [5], *J. communis* [6] and *J. oxycedrus* [7]. A binary mixture of **1**–**2** is found in *J. chinensis* Linn [8,9,10], *J. phoenicea* [11], *J. thurifera* var. *africana* [11], *J. foetidissima* [12], *J. sabina* [13], *Cryptomeria japonica* [14], *Platycladus orientalis* [15], *Sabina vulgaris* [16], *Podocarups imbricatus* BI [17], *Agathis vitiensis*, *A. macrophylla* and *A. lanceolata* [18], *Thuja occidentalis* L. [19], and *Hermas villosa* [20] whereas a mixture of **3**–**4** is found in *J. excelsa* [21]. *Trans*-communic acid (**1**) was isolated from *Entada abyssinica* [22], *Thujopsis dolabrata* [23,24,25], *Pinus luchuensis* [26], *Chamaecyparis obtusa* Endl. [27,28,29], *Thuja standishii* [30,31], *Araucaria angustifolia* [32], *Chamaecyparis formosensis* [33], *Porella navicularis* [34], *J. oxycedrus* [35], *J. drupaceae* Labill [36], *Sciadopitys verticillata* [37], *Fritillaria thunbergii* [38], *Cunninghamia unicanaliculata* var. *pyramidalis* [39], *Chromolaena collina* [40], *Cupressus sempervirens* [41,42], *J. communis* [43], *Chloranthus spicatus* [44], *Sabina vulgaris* Antoine [45], *Torreya jackii* [46], *Dacrydium pierrei* [47], *J. phoenicea* [48], *Calocedrus formosana* [49], *Fleischmannia multinervis* [50], *Cretan propolis* [51], *Libocedrus chevalieri* [52], *Pinus densiflora* [53], and *Mikania aff. jeffreyi* [54], *Chamaecyparis lawsoniana* [55]. *Cis*-communic acid (**2**) was detected in *Larix dahurica* [56], *Pseudotsuga wilsoniana* [57], and *Cladonia rangiferina* L. Web. [58].

*Myrceo*communic acid (**3**) was isolated from *Juniperus oxycedrus* [59]. Moreover the main component of diterpene acids in *Cunninghamia lanceolata* (Lamb.) Hook was 4-*epi*-*trans*-communic acid (**5**) [60]. Additionally polymers of **1** and of their derivatives have been found in resins of different *Agathis* species [61,62] and in sandarac resin [63].

Although these acids have been isolated from different parts of the plant (fruits, wood, bark, leaves, roots, *etc.*), they are mainly founded in leaves, fruits, and bark.

## 3. Biological Activity

The three communic acids **1**–**3** exhibited strong cytotoxic activity in a brine shrimp bioassay (LD_50_ 0.16 μg/mL) [46]. *Trans*-communic acid (**1**) and *cis*-communic acid (**2**) and plant extracts containing them were also active against different microorganisms such as *Staphylococcus aureus*, both standard ATCC strain and clinical isolates [55,64,65,66,67,68,69], *S. epidermidis* ATCC 12228 [70], *Aspergillus fumigatus* and *Candida albicans* [62]. Moreover, both acids have shown cytotoxic activity against BSC-1 cells [71]. Other activities described for **1** are: antimycobacterial (*Mycobacterium aurum*, *M. phlei*, *M. fortuitum* and *M. smegmatis*) [72], antitumoral [35,73], relaxant [74], hypolipidemic [75], testosterone 5α-reductase inhibitory [76], anti-inflammatory and antioxidant [77].

## 4. Chemical Reactivity

**Figure 2:**
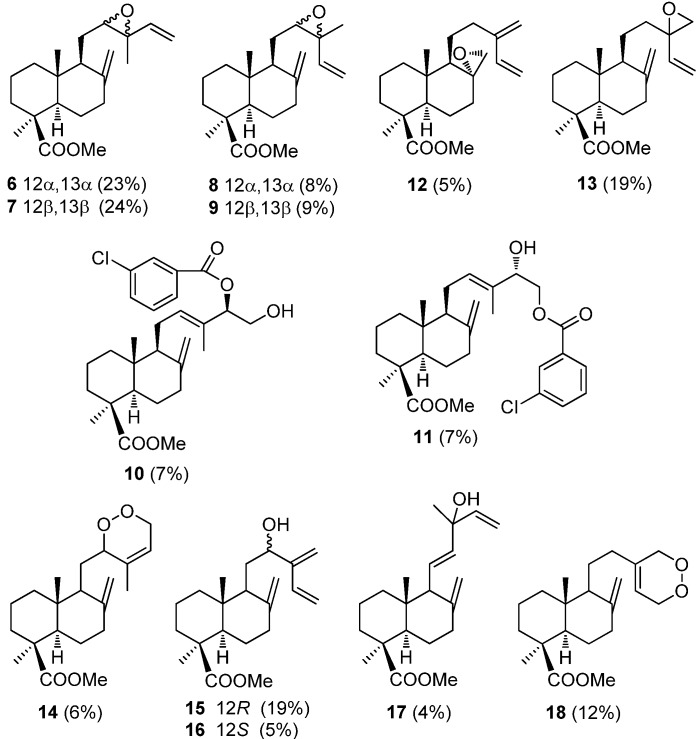
Structures of the products obtained by epoxidation and singlet oxygen oxidation of **1a**–**3a**.

Years ago, Pascual-Teresa *et al.* [78,79] described two studies based on the oxidation of the lateral chain of methyl esters of communic acids **1a**–**3a**. First, the functionalization of the side chain by selective epoxidation [78], and later the singlet oxygen addition [79] was studied. In both cases the relative reactivity of the three double bonds was determined for each compound. Epoxidation of **1a** and **2a** with *m*-chloroperbenzoic acid mainly afforded a mixture of 12,13-epopxy derivatives **6**–**9** together with a mixture of 14- and 15-*m*-chlorobenzoates **10**–**11** (Figure 2). The epoxidation of **3a** with *m*CPBA gave methyl (8*R*)-8,17-epoxy-8,17-dihydro*mirceo*communate (**12**, 5%) and methyl 13,16-epoxy-13,16-dihydro*mirceo*communate (**13**, 19%), recovering 24% yield of **3a**. This result indicates greater reactivity at the trisubstituted double bond for **1a** and **2a** and terminal double bond on the side chain for **3a**.

The singlet oxygen addition to **1a** led principally to the 12-hydroxyderivatives 12*R* (**15**, 19%) and 12*S* (**16**, 5%) together with minor proportions of the 12,15-dioxyderivative **14** (6%) and the tertiary alcohol **17** (4%), whereas in the case of **3a** afforded the only the 15,16-dioxyderivative **18** (12%).

Compound **1a** preferably underwent *ene*-reactions of the singlet oxygen on the trisubstituted double bond with *syn* stereospecificity, in accordance the with point established by Schulte-Elte [80]. Thus, the reaction produced mainly alcohols **15**–**17** and a minor proportion of the 12,15-dioxyderivative **14**, coming from a Diels-Alder reaction. In the case of methyl isocommunate **3a**, which does not have trisubstituted double bond and where the monosubstituted dienic system adopts the cisoid conformation with relative ease, the reaction that takes place with singlet oxygen is the Diels-Alder cycloaddition, slowly yielding a small amount of 15,16-dioxyderivative **18** due to the tendency of **3a** to polymerize.

**Scheme 1:**
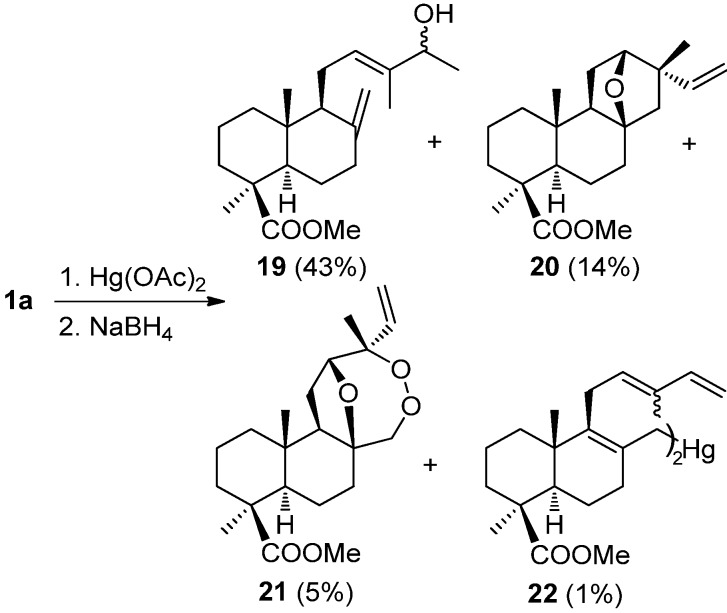
OM-OD reaction for compound **1a**. OD reaction with NaBH_4_.

Furthermore, another oxygenation procedure, *i.e.*, the oxymercuration-demercuration (OM-DM) reaction of methyl esters of *trans*- and *cis*-communic acids (**1a**–**2a**) was studied [81,82,83]. Treatment of **1a** with mercuric acetate (1:2) in THF/H_2_O and the subsequent reduction of mercurials with NaBH_4_ afforded compounds **19**–**22** (Scheme 1).

**Scheme 2:**
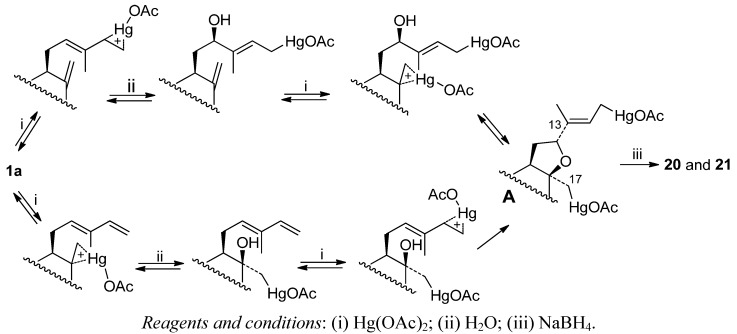
Mechanism of formation of compounds **20**, **21**.

Compound **19** is the product corresponding to the OM-DM at C14-C15 double bond. The formation mechanism of compounds **20**, **21** is shown in Scheme 2. The formation of tetrahydrofuran derivatives **20**–**21** from **1a**can be explained by two routes, both converging at intermediate **A** and evolving to **20**, **21**via radical processes. In the first route, **A** results from the formation of mercurinium ion on the 14,15 double bond, followed by 1,4 addition of water at C12, and heterocyclization by attack of the hydroxy group on the other mercurinium ion formed on the 8,17 double bond. In a second possible route, **A** is obtained by the hydration of the 8,17 double bond on the β face, followed by attack of the hydroxy group on carbon C12 on the mercurinium ion of the monosubstituted double bond. Both routes converge at the organomercurial **A**, whose reduction with NaBH_4_ in basic medium leads to the formation of a bis-radical intermediate, that by direct cyclization between carbons C13 and C17 originates **20**, and by reaction with atmospheric oxygen leads to **21**.

**Scheme 3:**
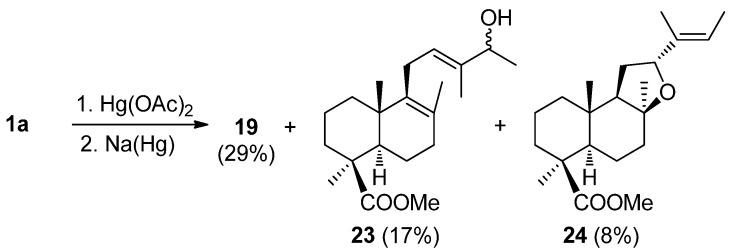
OM-OD reaction for compound **1a**. OD reaction with Na(Hg).

When the OM-OD reaction of compound **1a** was carried out using Na(Hg) as the demercuriating agent (Scheme 3), the products obtained were **19**, **23**–**24** and there was no evidence of the formation of either pimarane **20** or endoperoxide **21**. That is due to the fast reduction of the intermediate radicals coming from the corresponding type A organomercurials by sodium amalgam (Scheme 3).

**Scheme 4:**
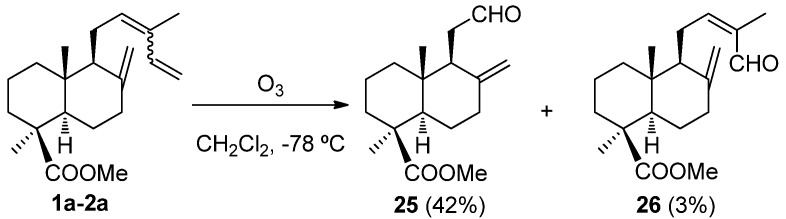
Ozonolysis of **1a**–**2a**.

Another interesting reaction from the synthetic point of view is the oxidative degradation of the C12,C13 double bond of either *cis*-, *trans*-communic acids or their methyl esters. This transformation opens the possibility of using them in the preparation of bioactive molecules. In order to find appropriate experimental conditions for regioselective oxidative cleavage of the C12,C13 double bond in presence of the 8(17) and 14,15 ones, two methods of double bond cleavage were tried on **1a**–**2a**: Ozonolysis and OsO_4_/NaIO_4_ treatment [84,85]. First, ozonolysis of **1a** was performed under different conditions, such as type of solvent (hexane, methanol, CH_2_Cl_2_), temperature (room temperature, 0 °C, −78 °C) and different ozone stream flows. Better selectivity towards the C12,C13 double bond degradation was observed when the reaction was carried out at −78 °C in CH_2_Cl_2_ yielding aldehyde-esters **25** and **26** (Scheme 4). The ozonolysis of isomer **2a** under the same conditions also led to preferential attack on the C12,C13 double bond giving rise to the same products (Scheme 4).

**Scheme 5:**
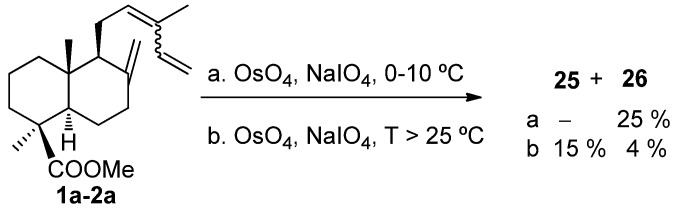
Oxidation of **1a**–**2a** with OsO_4_/NaIO_4_.

The outcome of the reaction of **1a**–**2a** with OsO_4_/NaIO_4_ is, however, strongly dependent on experimental conditions. Thus, when the temperature was kept at 0 °C to 10 °C, only **26** was detected, whereas mixtures of **25** and **26** were isolated when the temperature was 25 °C or higher (Scheme 5).

## 5. Use of Communic Acids as Starting Materials for the Synthesis of Compounds of High Added Value

**Scheme 6:**
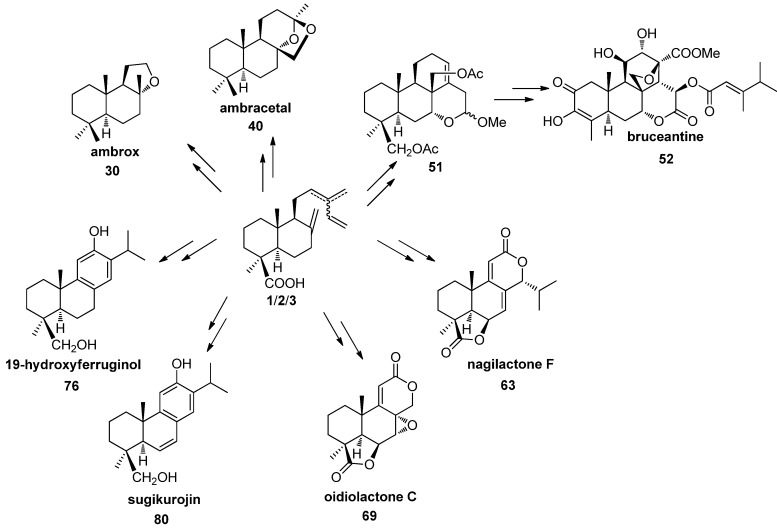
Compounds synthesized from communic acids **1**–**3**.

Communic acids 1–3 possess a labdane diterpene structure functionalised with a carboxylic group at C19, an exocyclic methylene at C8,C17 and a side chain dienic system appropriate for the preparation of a great variety of bioactive terpenoids, such as perfume fixatives [ambrox (**30**) and ambracetal (**40**)], antitumoral quassinoids [bruceantin (**52**)], antifungal podolactones [nagilactone F (**63**) and oidiolactone C (**69**)], and abietanes [19-hydroxyferruginol (**76**) a target for tolerance after transplant and in autoimmune diseases], and sugikurojin (**80**)] (Scheme 6).

**Scheme 7:**
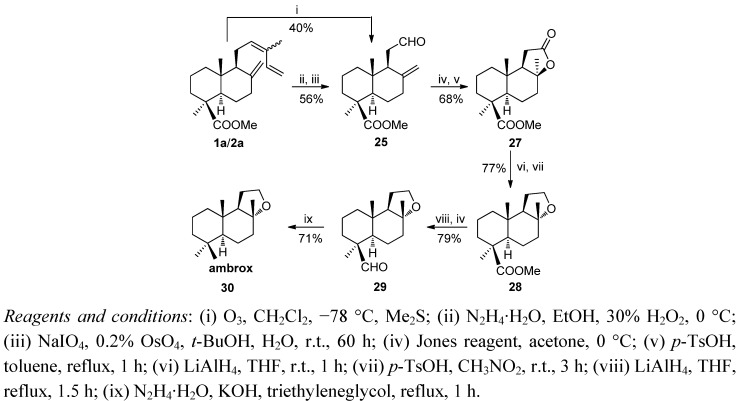
Synthesis of ambrox **30**.

**Scheme 8:**
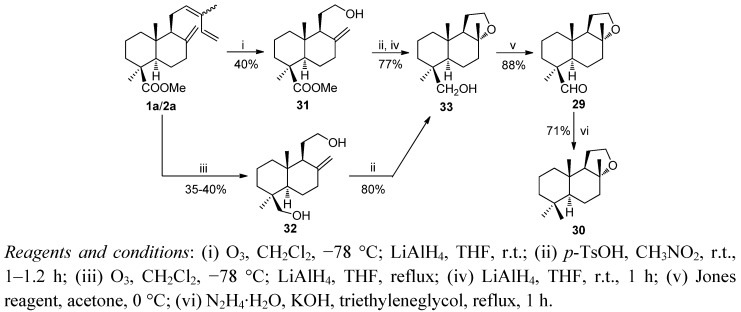
Synthesis of ambrox.

Ambrox (**30**) and ambracetal (**40**) are perfume fixatives with a powerful amber-type aroma. Their syntheses were carried out alternatively from methyl*trans*-communate (**1a**) or methyl *cis*-communate (**2a**) or a mixture of the two [86,87]. Two different routes to ambrox from **1a**/**2a** are showed in Scheme 7 and Scheme 8. The key steps of these syntheses are selective degradation of the side chains, stereoselective formation of the tetrahydrofuran ring and reduction of the axial methoxycarbonyl group. In the first synthesis the transformation of **1a** and/or **2a** to aldehyde **25** was done using two different methods: (a) carefully controlled ozonolysis of **1a** and/or **2a** at low temperature or (b) Δ^14^ selective hydrogenation with diimide, followed by a C12–C13 degradation of the resulting 14,15-hydrogenated derivative with OsO_4_/NaIO_4_. Oxidation of **25** with Jones reagent followed of cyclization with *p*-TsOH in toluene at reflux stereoselectively yielded the γ-lactone **27** with the most stable *cis* interannular linkage. Its reduction with LiAlH_4_ followed by kinetically controlled cyclization with *p*-TsOH/CH_3_NO_2_ at room temperature gave the tetrahydrofurane derivative **28** with the suitable *trans* stereochemistry. The conversion of the hindered methoxycarbonyl group into the methyl group was carried out in three steps by reduction of ester **28**, oxidation of the resulting alcohol to aldehyde **33** and finally reduction under Huang-Minlon conditions led to the target **30** (Scheme 7).

In the second route hydroxyolefin **31**, obtained by reductive ozonolysis from **1a**/**2a**, was treated with *p*-TsOH in CH_3_NO_2_ at room temperature and subsequently with LiAlH_4_ to give the alcohol **33**. Oxidation of **33** with Jones reagent led to the aldehyde **29** whose reduction under Huang Minlon conditions yielded ambrox (**30**). This route was improved and shortened by direct conversion of **1a**/**2a** into diol **32** by reductive ozonolysis followed of cyclization with *p*-TsOH in CH_3_NO_2_ to yield the alcohol **33** (Scheme 8).

**Scheme 9:**
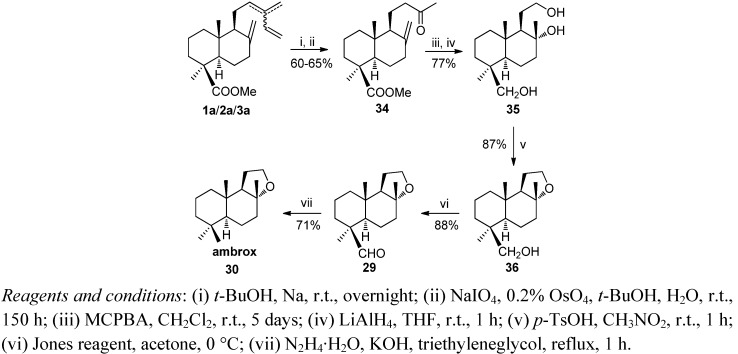
Synthesis of ambrox.

Mixtures of **1–3** from *Juniperus communis* fruits are of great interest because they are byproducts of gin manufacturing. Scheme 8 and Scheme 9 show the syntheses of ambrox and ambracetal from a mixture of methyl esters of **1**–**3**. The key intermediate in both processes is methyl ketone **34**. This compound was obtained efficiently by a chemoselective reduction of the dienic system of a mixture of **1a**–**3a** with Na/*t*-BuOH at room temperature and subsequent oxidation with OsO_4_/NaIO_4_. The transformation of **34** to trihydroxy derivative **35** was carried out by stereoselective epoxidation with *m*-CPBA at room temperature followed by reduction with LiAlH_4_ in THF at reflux. Stereo-selective cyclization of **35** with *p*-TsOH/CH_3_NO_2_ at room temperature led to **36**, which was transformed in ambrox **30** following the experimental procedure outlined in Scheme 7.

**Scheme 10:**
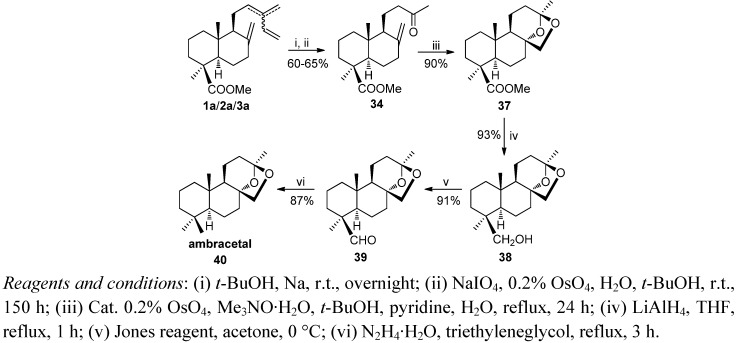
Synthesis of ambracetal.

For ambracetal (**40**) synthesis, treatment of methyl ketone **34** with a catalytic amount of OsO_4_ in a refluxing mixture of *t*-BuOH/pyridine/H_2_O and trimethylamine oxide as co-oxidant, afforded the tetracyclic ester **37** (Scheme 10). Conversion of the methoxycarbonyl group into the methyl group was carried out as shown in Scheme 6.

**Scheme 11:**
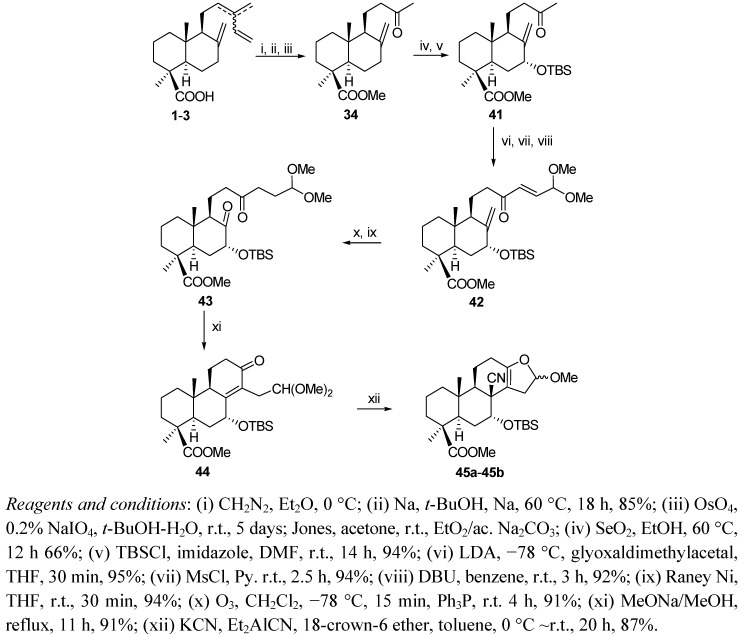
Synthesis of the tetracyclic intermediate **45**.

**Scheme 12:**
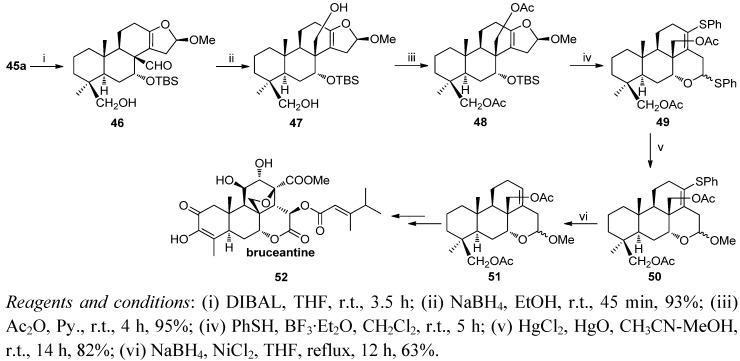
Synthesis of the intermediate **51** (precursor of bruceantin **52**).

An approach to compound **51**, an intermediate in the synthesis of the antitumor agent bruceantin (**52**) has been developed from the communic acids **1**–**3** (Scheme 11 and Scheme 12) [88] *via* the methyl ketone **34**.

Allylic oxidation of **34** at C7 with SeO_2_ at 60 °C and subsequent protection of the alcohol obtained with TBSCl yielded keto-ester **37** with high stereoselectivity. Subsequent condensation of the kinetic enolate of **41** with glyoxal dimethylacetal followed by mesylation and elimination with DBU led to the α,β-unsaturated ketone **42**. Chemoselective reduction of **42** with Raney nickel and subsequent ozonolysis afforded diketone **43**. At this point, an intramolecular aldol condensation gave the tricyclic ketone **44**, whose hydrocyanation with potassium cyanide, diethylaluminium cyanide and 18-crown-6 ether led with high stereoselectivity to an epimer mixture of acetals (**45a**–**b**) (6:1) (Scheme 11). Isomer **45a** was used to complete the synthetic sequence (Scheme 11). Thus, reduction of **45a**, first with DIBAL and then with NaBH_4_ afforded the diol **47**, which was acetylated yielding **48**. Exposure of **48** to thiophenol and boron trifluoride etherate in CH_2_Cl_2_ at room temperature yielded thioacetal **49**. This compound was obtained as an epimeric mixture and the thioether groups were sequentially removed with mercury (II) chloride and mercury oxide in acetonitrile/methanol (1:1) at room temperature. Compound **51** was finally obtained as an epimer mixture after reductive desulfurization of **50** using nickel boride (Scheme 12).

Podolactones are nor-or bisnorditerpenic compounds isolated mainly from different plants of the genus *Podocarpus* (family *Podocarpaceae*) [89], and filamentous fungi (*Oidodendrum truncatum* [90], *Aspergillus wentii* [91], and *Acrostalamus sp*. [92]). These molecules present a wide range of biological activity, including antitumoral, insecticidal, antifeedant, allelopathic, and fungicidal activities, special attention being paid to their the antifungal activity. In this regard, LL-Z1271α (**62**) and oidolactone C (**69**) exhibited potent antifungal activities [93,94].

**Scheme 13:**
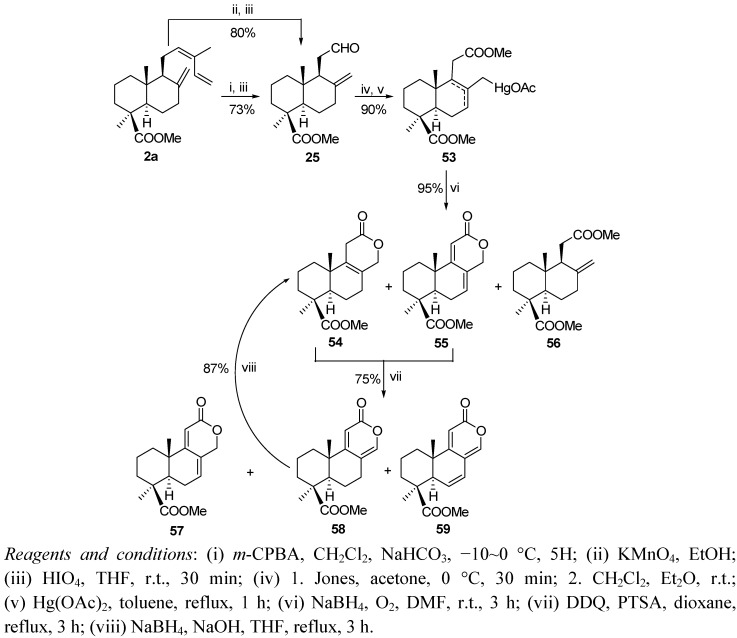
Synthesis of nagilactone F, LL-Z1271γ and LL-Z1271α.

**Scheme 14:**
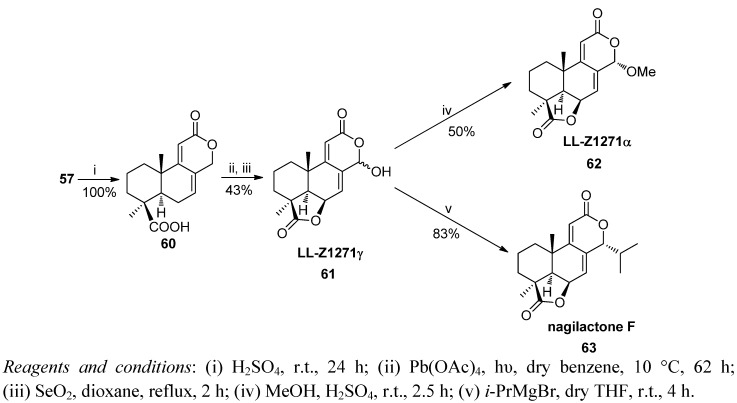
Synthesis of nagilactone F, LL-Z1271γ and LL-Z1271α.

Considering their interesting properties, the podolactones nagilactone F (**63**) and LL-Z1271α (**62**) have been synthesized from a mixture of **1**, **2** (Scheme 13 and Scheme 14) [95]. Now the key steps are a δ-lactonization in order to form the C ring, γ-lactonization and finally 14-hydroxylation.

The synthesis begins with the degradation of the side chain of the acids **1**,**2** by a different procedure to those previously described. Thus, oxidation with *m*-CPBA of the starting material and subsequent treatment of the crude product with HIO_4_ led to the aldehyde **25** with good yield (73%). Compound **25** was better obtained by potassium permanganate oxidation and subsequent periodic degradation (80%). Oxidation of **25** to a carboxylic acid and esterification with CH_2_N_2_ followed by treatment with mercuric acetate (2.0 equiv.) in toluene at reflux gave the derivative **53** as an 8:1 mixture (Δ^8^:Δ^7^). This mixture was reduced with NaBH_4_/DMF in the presence of an excess of bubbling O_2_, producing lactone **54** (75%), dienolide **55** (15%) and the starting product **56** (5%). This mixture was dehydrogenated with DDQ and PTSA to give an 8:3:1 mixture of **57**–**59**.

The methyl ester **57** was hydrolyzed almost quantitatively with concentrated sulphuric acid to obtain the acid **60**. The treatment of **60** with lead tetraacetate under argon atmosphere and ten with SeO_2_ led to the δ-hydroxylactone **61** permitting firstly γ-lactone closure and subsequently allylic oxidation at C14. Then the antibiotic LL-Z1271α (**62**) was prepared by treatment of **61** with methanol acidified with a drop of sulphuric acid. Moreover, treatment of **61** with isopropylmagnesium bromide at 0 °C yielded 83% of condensation products, being the most of the α isomer (90%), nagilactone F (**63**).

Related with the above-mentioned podolactone syntheses, the first synthesis of the antifungal oidiolactone C (**69**) was carried out from *trans*-communic acid (**1**) (Scheme 14) [96,97]. The key step of the synthesis is a new bislactonization reaction catalyzed by Pd(II), giving rise to the podolactone-type tetracyclic skeleton from a norlabdadienedioic acid. This synthetic scheme was also used by the authors to improve podolactone LL-Z1271α synthesis.

**Scheme 15:**
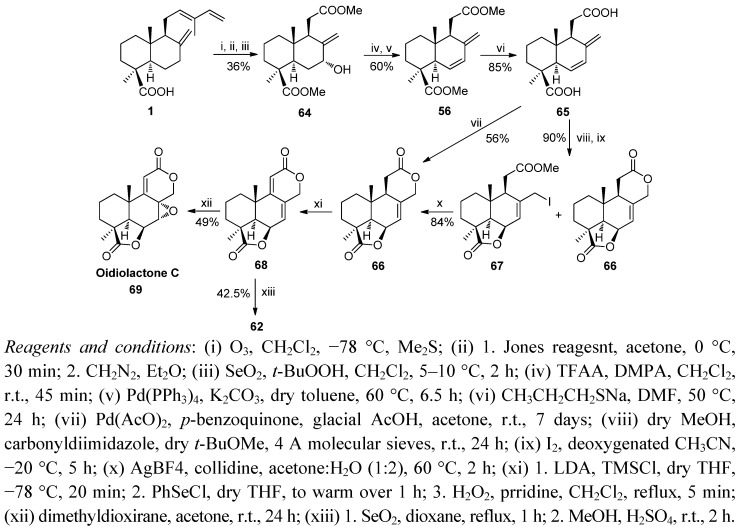
Synthesis of oidiolactone C and LL-Z1271α.

The selective ozonolysis of **1** and subsequent oxidation with Jones reagent, double esterification with diazomethane and allylic oxidation with SeO_2_/*t*-BuOOH yielded 36% of the hydroxydiester **64**. Elimination of the trifluoroacetate of **64** with Pd(PPh_3_)_4_ led to diene **56**, whose hydrolysis with sodium propanethiolate afforded diacid **65**. Two different procedures were employed to carry out the double lactonization. First, the selective methylation of the carboxyl group at C12 with MeOH in the presence of 1,1′-carbonyldiimidazole and then iodolactonization under Barrett’s conditions after strict deoxygenation of the reaction medium furnished the iodo derivative **67** (80% yield) along with a 20% yield of dilactone **66**. Iodo derivative **67** was exclusively converted in dilactone **66** by reaction with AgNO_3_/H_2_O/acetone (84% yield). Dilactone **66** was directly obtained from diacid **65** through a novel dilactonization process by treatment with substoichiometric Pd(II) (25%) and *p*-benzoquinone in a mixture of acetic acid and acetone as solvent (56%). The 9,11 double bond in diene-dilactone **68** was obtained, via the corresponding lithium enolate of **66** after adding phenylselenenyl chloride, and oxidation of the 11α-phenylseleno derivative to corresponding selenoxide by hydrogen peroxide with concomitant *syn*-elimination. Treatment of **68** with dimethyldioxirane afforded the natural oidiolactone C (**69**). Additionally, **62** was prepared by allylic oxidation as indicated in Scheme 15.

**Scheme 16:**
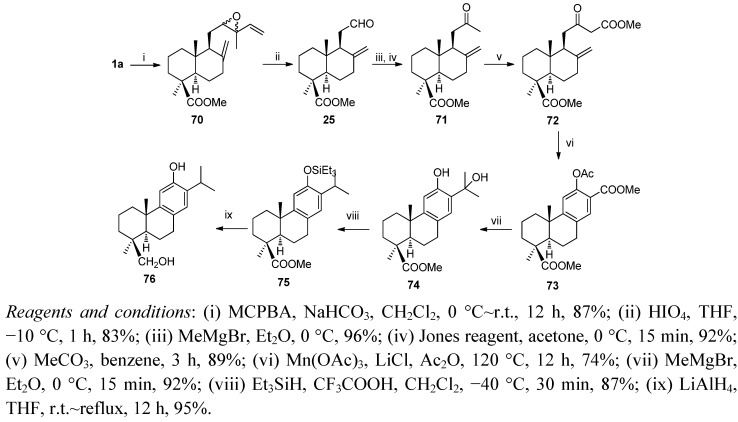
Synthesis of 19-hydroxyferruginol (**76**).

**Scheme 17:**
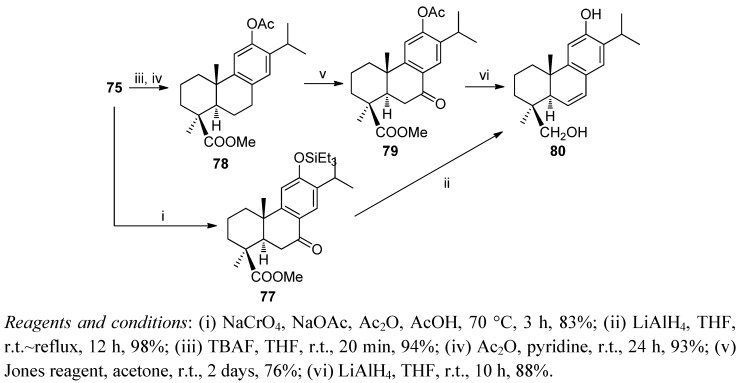
Synthesis of sugikurojin (**80**).

Synthesis of the phenol abietane diterpenes 19-hydroxyferruginol (**76**), isolated from *Podocarpus ferrugineus* [98], and sugikurojin A (**80**), isolated from *Cryptomeria japonica* [99], from *trans*-communic acid (**1**) is shown in Scheme 16 and Scheme 17, respectively [100]. The key steps of these procedures are the side chain degradation and the elaboration of the aromatic C ring by Mn(III) cyclization.

Epoxidation of ester **1a** by *m*CPBA followed by treatment with HIO_4_ in THF led to aldehyde **25**, whose treatment with MeMgBr and further oxidation with Jones reagent gave methylketone **71**. Reaction of **71** with Me_2_CO_3_ and NaH in benzene afforded the β-ketoester **72**. Treatment of **72** with Mn(OAc)_3_·2H_2_O (4.0 equiv.) and LiCl (3.0 equiv.) in Ac_2_O at 120 °C for 12 h led to the methyl *O*-acetyl salicylate **73** (74% yield). Transformation of **73** in abietane **74** was carried out by the addition of MeMgBr in excess. When this compound was treated with Et_3_SiH and CF_3_COOH was obtained silylether **75**, whose treatment with LiAlH_4_ in THF at reflux afforded 19-hydroxyferruginol (**76**) (Scheme 16).

Heating of silylether **75** with Na_2_CrO_4_ and NaOAc in Ac_2_O-AcOH led to 7-oxoderivative **77**. Compound **77** was refluxed with LiAlH_4_ in THF giving sugikurojin A (**80**). An alternative route to compound **80** from **75** involved the removal of the silyl group and further acetylation and oxidation to obtain ketone **79**, which was then transformed into **80** (Scheme 17).

## 6. Conclusions

This paper reveals the occurrence of the communic acids in fam. *Cupresaceae* especially in genus *Juniperus*. Furthermore they constitute appropriate building blocks for the efficient preparation of interesting bioactive natural products as ambrox, nagilactone F, bruceantin, 19-hydroxyferruginol and others.
